# Instrumentation failure following pediatric spine deformity growth-sparing surgery using traditional growing rods or vertical expandable prosthetic titanium ribs

**DOI:** 10.1186/s12891-024-07211-9

**Published:** 2024-02-08

**Authors:** Noriaki Yokogawa, Satoru Demura, Tetsuya Ohara, Ryoji Tauchi, Kosuke Takimura, Haruhisa Yanagida, Toru Yamaguchi, Kota Watanabe, Satoshi Suzuki, Koki Uno, Teppei Suzuki, Kei Watanabe, Toshiaki Kotani, Keita Nakayama, Norihiro Oku, Yuki Taniguchi, Hideki Murakami, Takuya Yamamoto, Ichiro Kawamura, Katsushi Takeshita, Ryo Sugawara, Ichiro Kikkawa, Noriaki Kawakami

**Affiliations:** 1 Department of Orthopaedic Surgery, Graduate School of Medical Sciences, Kanazawa University, 13-1 Takara-machi, Kanazawa, 920-8641 Ishikawa Japan; 2 Department of Orthopaedic Surgery, Meijo Hospital, Aichi, Japan; 3 Department of Orthopaedic and Spine Surgery, Fukuoka Children’s Hospital, Fukuoka, Japan; 4 Department of Orthopaedic Surgery, Keio University School of Medicine, Tokyo, Japan; 5 Department of Orthopaedic Surgery, National Hospital Organization, Kobe Medical Center, Hyogo, Japan; 6 Department of Orthopaedic Surgery, Niigata University, Niigata, Japan; 7 Department of Orthopaedic Surgery, Seirei Sakura Citizen Hospital, Chiba, Japan; 8 Department of Orthopaedic Surgery, The University of Tokyo, Tokyo, Japan; 9 Department of Orthopaedic Surgery, Iwate Medical University, Iwate, Japan; 10 Department of Orthopaedic Surgery, Kagoshima Red Cross Hospital, Kagoshima, Japan; 11 Department of Orthopaedic Surgery, Kagoshima University, Kagoshima, Japan; 12 Department of Orthopaedic Surgery, Jichi Medical University, Tochigi, Japan; 13 Department of Pediatric Orthopedics, Jichi Children’s Medical Center, Tochigi, Japan; 14 Department of Orthopaedic Surgery, Ichinomiya Nishi Hospital, Aichi, Japan

**Keywords:** Growth-sparing surgery, Instrumentation failure, Pediatric spine deformity, Traditional growing rods, Vertical expandable prosthetic titanium ribs

## Abstract

**Background:**

Instrumentation failure (IF) is a major complication associated with growth-sparing surgery for pediatric spinal deformities; however, studies focusing on IF following each surgical procedure are lacking. We aimed to evaluate the incidence, timing, and rates of unplanned return to the operating room (UPROR) associated with IF following each surgical procedure in growth-sparing surgeries using traditional growing rods (TGRs) and vertical expandable prosthetic titanium ribs (VEPTRs).

**Methods:**

We reviewed 1,139 surgical procedures documented in a Japanese multicenter database from 2015 to 2017. Of these, 544 TGR and 455 VEPTR procedures were included for evaluation on a per-surgery basis. IF was defined as the occurrence of an implant-related complication requiring revision surgery.

**Results:**

The surgery-based incidences of IF requiring revision surgery in the TGR and VEPTR groups were 4.3% and 4.0%, respectively, with no significant intergroup difference. Remarkably, there was a negative correlation between IF incidence per surgical procedure and the number of lengthening surgeries in both groups. In addition, rod breakage in the TGR group and anchor-related complications in the VEPTR group tended to occur relatively early in the treatment course. The surgery-based rates of UPROR due to IF in the TGR and VEPTR groups were 2.0% and 1.5%, respectively, showing no statistically significant difference.

**Conclusions:**

We found that IF, such as anchor related-complications and rod breakage, occurs more frequently earlier in the course of lengthening surgeries. This finding may help in patient counseling and highlights the importance of close postoperative follow-up to detect IF and improve outcomes.

## Background

Growth-sparing surgery is commonly performed to correct pediatric scoliosis and other spinal deformities while maintaining spinal growth and maximizing thoracic volume and pulmonary function. Although growth-sparing surgery typically produces favorable outcomes, its high complication rate due to the need to undergo multiple surgeries at a very young age presents a problem [1]. Complications related to growth-sparing surgery often necessitate an unplanned return to the operating room (UPROR), which can lead to poor outcomes [2, 3].

Previous studies have indicated that a higher number of lengthening surgeries is associated with increased complications [4, 5]; therefore, efforts have been made to delay the interval until index surgery or prolong the intervals between lengthening surgeries to reduce the number of lengthening surgeries and, thus, the risk of complications. However, these studies were patient-based and related to overall complications. Major complications associated with growth-sparing surgery include wound infection and delayed wound healing, anesthesia-related and other systemic complications, and instrumentation failure (IF); each of these complications should be evaluated separately. While IF is considered the most frequent of these complications [1], there is a lack of studies focusing on IF following each lengthening surgery procedure. Therefore, this study was conducted on a per-surgery basis to determine at what point during the series of growth-sparing surgical processes IF is more likely to occur and at what point revision surgery following IF is performed. Using a multicenter research database, we investigated IF in the following surgical approaches: traditional growing rods (TGRs) and vertical expandable prosthetic titanium ribs (VEPTRs), as these are the main surgical approaches used for pediatric spinal deformities in Japan.

## Methods

We retrospectively reviewed the Japan Spinal Deformity Institute database, which registers pediatric spinal deformity surgeries performed at multiple institutions in Japan, between January 2015 and December 2017. Ethics approval was obtained from the ethics committees of Kanazawa University and the other participating institutions (ethical approval number: 2016 − 337). The need for informed consent was waived by the ethics committees of Kanazawa University and the other participating institutions because of the retrospective nature of the study.

As this study was conducted on a per-surgery basis, information concerning surgical procedures relating to TGRs and VEPTRs was extracted from the database. Final fixations, unplanned surgeries (including revisions), and surgeries with missing data were excluded, while planned index and lengthening surgeries followed up until the subsequent surgery or for a minimum of 2 years were included. IF was defined as any implant-related complication requiring revision surgery. The following data were extracted for inclusion in the analysis: incidence of IF per surgical procedure, type of IF (including anchor loosening and dislocation, rod or connector breakage, implant protrusion, and proximal junctional kyphosis), timing of IF, and timing of revision surgery due to IF. IFs were evaluated on routine radiographs before the scheduled next surgery. Proximal junctional kyphosis was defined as kyphosis in which the angle between the fixed superior end vertebra and its two cephalad vertebrae is > 10° and the difference from the preoperative angle is > 10°, according to the criteria of Glattes [6]. The timing of revision surgery was categorized into three groups: electively and concurrent with the next scheduled surgery, consequently and concurrent with the next scheduled surgery, and UPROR. When a revision surgery was performed concurrent with the next scheduled surgery, we defined “Consequential” as cases in which UPROR was usually considered necessary but was consequently treated as such and “Elective” as cases in which performing a standby surgery was deemed possible.

In the statistical analysis, the incidences of IF per surgical procedure and UPROR for IF were compared between the TGR and VEPTR groups using the chi-square test. The Cochran–Armitage test was used to analyze the association between the performed lengthening surgeries and the incidence of IF to examine after which lengthening surgery IFs are more likely to occur. Statistical significance was set at *P* < 0.05. Statistical analyses were performed using SPSS software version 23 (IBM Corp., Armonk, NY, USA) and JMP software version 16.1.0 (SAS Institute, Cary, NC, USA).

## Results

A total of 1,139 growth-sparing surgeries from 11 institutions were registered in the Japan Spinal Deformity Institute database from 2015 to 2017. Of these, 576 procedures in 152 patients were registered as TGRs and 531 in 137 patients as VEPTRs, of which 58 and 31 procedures could clearly be classified as index surgeries and 486 and 424 as lengthening surgeries, respectively. In the TGR group, 83 patients were boys and 69 were girls, whereas in the VEPTR group, 65 were boys and 72 were girls. The etiologies were idiopathic, congenital, neuromuscular, and syndromic in 25, 35, 25, and 67 cases in the TGR group and 2, 99, 14, and 22 cases in the VEPTR group, respectively. The average age at the time of surgery was 9.8 years in the TGR group and 8.4 years in the VEPTR group.

The incidence of IF per surgical procedure is presented in Table 1. The incidence of IF was 4.3% in the TGR group and 4.0% in the VEPTR group, with no statistically significant intergroup difference (*P* = 0.72). Anchor loosening and dislocation were the most common complications in the TGR group (62.5%), followed by rod breakage (33.3%). In contrast, anchor loosening and dislocation were the most common (61.1%) complications in the VEPTR group, followed by rod breakage and implant protrusion (both 16.7%). The level of anchor loosening and dislocation varied in the VEPTR group, while it was isolated to the cephalic level in the TGR group.

**Table 1:** Incidence of instrumentation failure

	TGRs	VEPTRs	*P*
No. of surgeries	544	455	-
No. of IFs	24	18	-
Incidence of IF, %	4.3/surgery	4.0/surgery	0.72
Details of IF, n (%)	- Anchor loosening/dislocation: 15 (62.5) (all cephalad) - Rod breakage: 8 (33.3) - Connector breakage: 1 (4.2)	- Anchor loosening/dislocation: 11 (61.1) (cephalad: 6, caudal: 4, unknown: 1) - Rod breakage: 3 (16.7) - Implant protrusion: 3 (16.7) - Proximal junctional kyphosis: 1 (5.6)	-

IF: instrumentation failure, TGRs: traditional growing rods, VEPTRs: vertical expandable prosthetic titanium ribs

The timing of IF occurrence in the TGR group is presented in Table 2. The incidence rates of IF after index and lengthening surgeries were 6.9% and 4.1%, respectively. Remarkably, the incidence of IF tended to decrease as the number of lengthening surgeries increased. In the analysis of each IF type, rod breakage and the number of lengthening surgeries were negatively correlated, while no significant correlation was observed for anchor-related complications.

The timing of IF occurrence in the VEPTR group is shown in Table 3. The incidence rates of IF for index and lengthening surgeries were 6.5% and 3.8%, respectively. Similar to that in the TGR group, the incidence of IF in the VEPTR group tended to decrease with an increasing number of lengthening surgeries. In the analysis of each IF type, there was a negative correlation between the occurrence of anchor-related complications and the number of lengthening surgeries, while there were no significant correlations for rod breakage and implant protrusion.

**Table 2:** Timing of instrumentation failure in traditional growing rod technique

	Index surgery	Lengthening surgery				*P* for trend
		1st–3rd	4th–6th	7th–9th	≥ 10th	
No. of surgeries	58	173	154	99	60	-
No. of IFs (%)	4 (6.9)	11 (6.4)	7 (4.5)	2 (2.0)	0 (0)	0.01*
No. of anchor loosenings/dislocations (%)	4 (6.9)	5 (2.9)	4 (2.6)	2 (2.0)	0 (0)	0.23
No. of rod breakages (%)	0 (0)	5 (2.9)	3 (1.9)	0 (0)	0 (0)	0.04*
No. of connector breakages (%)	0 (0)	1 (0.6)	0 (0)	0 (0)	0 (0)	0.28

IF: instrumentation failure

*Statistically significant association between the number of lengthening surgeries performed and the surgery-based incidence of IF in the Cochran–Armitage test

**Table 3:** Timing of instrumentation failure in vertical expandable prosthetic titanium rib technique

	Index surgery	Lengthening surgery				*P* for trend
		1st–3rd	4th–6th	7th–9th	≥ 10th	
No. of surgeries	31	167	133	85	39	-
No. of IFs (%)	2 (6.5)	12 (7.2)	2 (1.5)	2 (2.4)	0 (0)	0.01*
No. of anchor loosenings/dislocations (%)	2 (6.5)	8 (4.8)	1 (0.8)	0 (0)	0 (0)	< 0.01*
No. of rod breakages (%)	0 (0)	1 (0.6)	0 (0)	2 (2.4)	0 (0)	0.55
No. of implant protrusions (%)	0 (0)	2 (1.2)	1 (0.8)	0 (0)	0 (0)	0.24
No. of proximal junctional kyphoses (%)	0 (0)	1 (0.6)	0 (0)	0 (0)	0 (0)	0.31

IF: instrumentation failure

*Statistically significant association between the number of lengthening surgeries performed and the surgery-based incidence of IF in the Cochran–Armitage test

The timings of revision surgery in the TGR and VEPTR groups are presented in Tables 4 and 5, respectively. Overall, nearly half of the cases with IF required UPROR; the UPROR rate was 2.0% in the TGR group and 1.5% in the VEPTR group, showing no statistically significant difference (*P* = 0.57). The timing of revision surgery was largely dependent on IF type; rod breakage in both groups frequently required UPROR, whereas revision procedures for anchor-related complications were usually performed at the next scheduled surgery. However, approximately half of the revision surgeries performed in conjunction with scheduled surgeries were consequential rather than elective.

**Table 4:** Timing of revision surgery following instrumentation failure in traditional growing rod technique

Type of instrumentation failure	Timing of revision surgery		
	Next scheduled surgery: elective, n (%)	Next scheduled surgery: consequential, n (%)	Unplanned return to the operating room, n (%)
Anchor loosening/dislocation	5 (0.9)	6 (1.1)	4 (0.7)
Rod breakage	0 (0)	1 (0.2)	7 (1.3)
Connector breakage	0 (0)	1 (0.2)	0 (0)
Total	5 (0.9)	8 (1.5)	11 (2.0)

**Table 5:** Timing of revision surgery following instrumentation failure in vertical expandable prosthetic titanium rib technique

Types of instrumentation failure	Timing of revision surgery		
	Next scheduled surgery: elective, n (%)	Next scheduled surgery: consequential, n (%)	Unplanned return to the operating room, n (%)
Anchor loosening/dislocation	3 (0.7)	4 (0.9)	4 (0.9)
Rod breakage	0 (0)	1 (0.2)	2 (0.4)
Implant protrusion	2 (0.4)	0 (0)	1 (0.2)
Proximal junctional kyphosis	1 (0.2)	0 (0)	0 (0)
Total	6 (1.3)	5 (1.1)	7 (1.5)

## Discussion

There have been many reports on complication rates of growth-sparing surgeries for pediatric spinal deformities, with the rate per patient ranging from 19 to 208% in TGRs and 17–226% in VEPTRs [1–5, 7–22]. Various factors may contribute to this wide variation, including differences in patient and disease backgrounds, inconsistent definitions of complications, and differences in the number of lengthening surgeries per patient and their intervals. Therefore, it is important to determine not only the complication rate per patient but also per surgical procedure and the timing of complication occurrences. Thus, we used a surgery-based approach focused on IF, which is the most frequent complication, and only included cases of IF requiring revision surgery.

In a few studies where data on surgery-based complication rates are available, the reported complication rates for TGRs range from 22 to 37% per surgery, and the IF rates range from 12 to 19% per surgery [4, 5, 7, 8]. Although direct comparisons may not be appropriate, the IF incidence per surgical procedure in the TGR group of this study was relatively lower than those previously reported. This may be partly attributed to the fact that the present study only included cases of IF requiring revision surgery to ensure that IF was clearly defined. Additionally, this study included surgeries that were performed relatively recently, and the incidence of IF in growth-sparing surgeries for pediatric spinal deformities has probably been steadily decreasing owing to accumulated data over time relating to the type, number, and level of the anchors, as well as the material and number of the rods for successful outcomes [5, 9–14].

The reported complication rates for VEPTRs range from 13 to 48% per surgery, while the IF rates range from 4 to 18% per surgery [15–17]. The incidence of IF in the present cohort was lower than those of previous studies; this may be attributed to the same cause as the low incidence in TGRs. In contrast, the results of this study were consistent with those of a previous study comparing TGRs and VEPTRs, which found no significant difference in IF incidence between the two surgical methods [18]. Magnetically controlled growth rods, which eliminate the need for multiple lengthening surgeries and the accompanying general anesthesia and skin incisions, have recently been implemented in Europe and the United States; however, the complication rates were reported not to be lower than those of TGRs [7, 19, 20]. Even though the use of magnetically controlled growth rods is associated with a low risk of wound infection, the risk of implant-related problems and UPROR remains high. Thus, it is assumed that growth-sparing surgeries based on the concept of multi-lengthening would have comparable rates of implant-related complications.

In the present study, anchor-related complications accounted for the highest number of IFs for both TGRs and VEPTRs; however, the level of occurrence was distinctive, with all anchor-related complications in TGRs appearing to be cephalic. In a study by Liang et al. of complications in the TGR technique, dislodged implants were the most common IF (76%), with 92% of these occurring at the cephalic level [21]. Although the authors suggested a possible association with a persistent asymmetric force load on the cephalic anchor, the mechanism remains unclear and requires further investigation.

Historically, the complication rate per patient in growth-sparing surgery generally increases with the number of lengthening procedures. Watanabe et al. reported that six or more lengthening procedures was an independent risk factor for complications in the TGR procedure [4]. Additionally, Bess et al. noted that the risk of complications increased by 24% for each additional TGR procedure [5]. In contrast, the present study demonstrated that the incidence of IF per surgical procedure decreased with an increasing number of lengthening surgeries for both methods. This may reflect the differing nature of the complications; wound- and anesthesia-related complications other than IF have been reported to be more likely to occur with a greater number of lengthening surgeries [22–24]. Furthermore, the decrease in the incidence of IF is probably attributed to the stabilization of the anchor over time, as well as autofusion impairing spinal flexibility and reducing the force load on the implant. IF may also cause withdrawal from the course of growth-sparing surgery, which may be another contributing factor. Nevertheless, IF, such as anchor related-complications and rod breakage, occurs more frequently earlier in the course of lengthening surgeries. Thus, a careful explanation of these points to the patient and guardian is important to prevent discouragement due to unexpected complications, and close postoperative follow-up for IF should be performed.

A review article has reported the unscheduled surgery rates per surgical procedure to be 3–36% in TGRs and 3–17% in VEPTRs [1]. These reoperation rates vary widely in their definitions; therefore, in this study, revision surgeries were divided into three different time categories: electively concurrent with the next scheduled surgery, consequentially concurrent with the next scheduled surgery, and UPROR. The UPROR rates for IF in this study were relatively low; however, revision surgeries were consequentially performed in conjunction with scheduled surgery in several cases, probably owing to delayed IF detection. This again highlights the importance of careful postoperative observation.

This study is limited by the fact that only data from surgeries performed over a 3-year period were available. Consequently, we could not assess the entire course of each patient, from the index surgery to the final fixation. We were also unable to examine the association between IF and patient-specific factors, such as background and radiographic parameters. Therefore, to maximize the utilization of existing data while eliminating as much as possible the influence of this limitation, this study was conducted not on a per-patient basis, but on a per-surgery basis, focusing on the incidence and timing of IF and related revision surgeries. However, it is certainly important to investigate patient demographics and radiographic parameters and their relationship to IF. We are currently in the process of accumulating data for a patient-based data analysis covering a series of treatment courses for growth sparing surgery. Furthermore, this study has a retrospective design, and the surgical indications and follow-up protocols may differ across institutions. In addition, IF that did not require revision surgery was not evaluated. Although further research is necessary to support our evidence, we believe that the findings of this study are unprecedented and significant as this is the first multicenter study to examine the incidence and timing of IF and related revision surgeries in cases where TGRs and VEPTRs were used.

## Conclusions

There were no significant differences between the TGR and VEPTR methods to treat pediatric spinal deformity in terms of the surgery-based incidence of IF requiring revision surgery (4.3% vs. 4.0%) and UPROR due to IF (2.0% vs. 1.5%). The most remarkable finding of this study was the negative correlation between IF incidence per surgical procedure and the number of lengthening surgeries in both methods. We believe that the findings of this study will help guide patient counseling; they also highlight the importance of close postoperative follow-up to detect instrumentation failure in a timely manner and promote improved patient outcomes.

## Data Availability

The study data and details of materials used may be made available upon reasonable request by sending an e-mail to the first author.

## Abbreviations

IF: Instrumentation failure
TGRs: Traditional growing rods
UPROR: Unplanned return to the operating room
VEPTRs: Vertical expandable prosthetic titanium ribs
